# Predictors of health-related quality of life in stroke patients after neurological inpatient rehabilitation: a prospective study

**DOI:** 10.1186/s12955-015-0258-9

**Published:** 2015-05-14

**Authors:** Mirjam Katona, Ralf Schmidt, Wilfried Schupp, Elmar Graessel

**Affiliations:** Centre of Health Services Research in Medicine, Department of Psychiatry and Psychotherapy, Friedrich-Alexander-University Erlangen-Nuernberg, Schwabachanlage 6, 91054 Erlangen, Germany; Clinic for Specialised In- and Outpatient Rehabilitation Medicine Herzogenaurach, Department of Neurology and Neuropsychology, In der Reuth 1, 91074 Herzogenaurach, Germany

**Keywords:** Health-related quality of life, EQ-5D, Stroke, Rehabilitation, Predictors, Prospective study

## Abstract

**Background:**

The goal of the study was to investigate the long-term course of health-related quality of life (HRQoL) in stroke survivors during and up to 2.5 years after inpatient neurological rehabilitation and to identify predictors of HRQoL.

**Methods:**

HRQoL was determined in 152 stroke survivors in a single-centre prospective cohort study at four time points: upon admission to inpatient rehabilitation, at discharge, and one and 2.5 years after discharge. Their HRQoL was determined by administering the EQ-5D at all four measurement points. During inpatient rehabilitation, the SF-36 was administered in addition to the EQ-5D. Predictors were identified through multiple regression analysis.

**Results:**

During inpatient rehabilitation, the “European Index” of the EQ-5D rose significantly (*p* < 0.001) from 45.4 to 66.7. The change in HRQoL on the SF-36 was convergent. The HRQoL of the stroke patients living at home remained at the same level for 2.5 years following discharge. In the multiple regression analysis, the EQ-5D Index at discharge (*p* = 0.049), the risk of falls as defined by Runge and Rehfeld (*p* = 0.001), and the change in emotional quality of life on the SF-36 during inpatient rehabilitation (*p* = 0.048) predicted HRQoL 2.5 years following discharge.

**Conclusion:**

On the basis of our results, we conclude that the long-term health-related quality of life of stroke survivors can be positively influenced by reducing the risk of falls and improving emotional well-being during neurological inpatient rehabilitation.

**Electronic supplementary material:**

The online version of this article (doi:10.1186/s12955-015-0258-9) contains supplementary material, which is available to authorized users.

## Introduction

Health is a state of complete physical, mental, and social well-being and not merely the absence of disease or infirmity [1]. This statement defines health-related quality of life (HRQoL) as a construct that focusses on the respondent’s subjective perception and consists of physical, mental, and social dimensions [2]. Self-reported HRQoL is increasing in importance as an outcome variable, particularly in many different chronic conditions [3–5].

The first aim of this study was to depict the course of HRQoL in stroke survivors by first measuring it at admission to inpatient neurological rehabilitation and measuring it several times until 2.5 years following discharge using two established measures, the EQ-5D and the SF-36. Comparisons with data from other European countries are subject to certain limitations. In Sweden, Algurén et al. investigated stroke patients from stroke units for up to a maximum of 1 year post-stroke [6]. In Great Britain, there are longitudinal data for the EQ-5D for 1 month to 5 years post-stroke. However, the results of both of these studies were not linked to rehabilitation data [7].

A long-term treatment goal in stroke survivors is to achieve HRQoL scores that are as high as possible. Therefore, variables that predict HRQoL are of special interest. The review article by Carod-Artal et al. provides an overview of the predictors of HRQoL in stroke survivors reported by longitudinal studies. These are age, sex, stroke severity, physical impairment, functional status, and mental impairment. However, there are substantial methodological differences between the eight studies that were analysed, starting with the nature of the data source (i.e. the patient data came from stroke units, rehabilitation units, population-based studies, and stroke registers). The numbers of cases that were included ranged from 77 to 397. The times at which HRQoL was measured ranged in most studies from 3 to 12 months post-stroke. In the analyses of HRQoL predictors, each of these studies used different socio-demographic or clinical parameters [8]. Only one study used the EQ-5D to determine HRQoL as a cross-sectional measure after 4 years [9]. The other studies analysed in this review used other measures (e.g. the SF-36 or the Stroke Impact Scale) [8]. Therefore, it was not possible to obtain consistent results.

The second and most important aim of our study was to establish which socio-demographic, clinical, and stroke-related parameters measured during the period of inpatient rehabilitation would predict quality of life as determined by the EQ-5D 2.5 years after discharge. For the first time, general geriatric risk factors such as the risk of falls, the risk of malnutrition, and the effect of comorbidities and data on change during the “inpatient rehabilitation” intervention were included.

## Materials and methods

### Study design

Data were obtained in a single-centre prospective cohort study that followed stroke patients for a period of 2.5 years after their discharge from neurological inpatient rehabilitation.

In Germany, most stroke survivors with functional deficits after acute treatment are offered neurological inpatient or outpatient rehabilitation. This study included all stroke survivors who underwent neurological inpatient rehabilitation at the Fachklinik Herzogenaurach (Clinic for In- and Outpatient Rehabilitation Medicine providing medical care to patients from the metropolitan areas of Nuremberg, Fuerth, and Erlangen in Bavaria, Germany–including urban and rural regions) and who met the following inclusion criteria: (a) stroke according to the WHO (World Health Organisation) definition; (b) moderate to severe functional deficits; (c) living at home before the stroke; and (d) informed consent. An additional file shows these criteria in detail (see Additional file 1: Figure S1). As the study focussed on follow-ups with patients who had deficits in their activities of daily living (ADL) functioning (see b), we included only patients in phase B (early neurological rehabilitation) or C (comprehensive neurological rehabilitation) according to the German model of phases in neurological rehabilitation [10]. Patients in phase D were excluded because they had only mild functional deficits upon admission. A total of 25 (11 %) patients dropped out during the inpatient rehabilitation for various reasons, and 22 (8 %) patients did not provide informed consent to participate in this study. Of the 204 patients who were discharged, 17 were institutionalised, and 35 died within the 2.5 years (see Additional file 1: Figure S1). Consequently, 152 patients were followed-up.

The study was reviewed and approved by the Ethics Committee of the Faculty of Medicine of the Friedrich-Alexander-University of Erlangen-Nuernberg (No. 3465).

### Data collection

In addition to the patients’ age and sex, the following clinical data were collected during inpatient rehabilitation: stroke aetiology, stroke subtype and side of lesion, comorbid conditions, ADL functioning, body mass index (BMI), nutritional status, risk of falls, and health-related quality of life. In order to measure functional change during inpatient rehabilitation, the scales for ADL functioning and health-related quality of life were administered twice (SF-36 and EQ-5D upon admission and at discharge).

A study nurse conducted telephone interviews with the patients or with their caregivers after 1 and 2.5 years to determine the patients’ situations (i.e. whether they were living at home, institutionalised, or deceased) and to record their health-related quality of life (EQ-5D). After three unanswered phone calls (9 cases, 4 %), the patient’s living status or new address was obtained from the local residents’ registration office. In this way, we established that 152 patients were still living at home 2.5 years after discharge. In 10 cases, the patient or caregiver could not be reached by phone. Therefore, the EQ-5D was completed by 142 patients.

### Instruments

The WHO definition of stroke was used to categorise stroke subtypes: ischemic stroke, intracerebral haemorrhage, subarachnoid haemorrhage, cerebral, or sinus venous thrombosis. The aetiological classification of the ischemic stroke is based on the TOAST classification scheme [11]. Comorbidities were adjusted by means of the Charlson Index [12], which assigns weights to specific comorbidities in accordance with their 1-year mortality risk (e.g. one point for cardiac infarction and six points for metastasising tumour). The higher the score, the higher the mortality risk.

The Barthel Index (BI) [13] and the Extended Barthel Index (EBI) [14] were used to assess the patients’ functional independence and cognitive skills related to activities of daily living. Seven items referring to early rehabilitation (e.g. dysphagia) were assessed by means of the Early Rehabilitation Barthel Index (ERBI) [15]. Nutritional parameters, severity of the disease, and age were taken into account by the Nutritional Risk Score [16]. Higher scores (0–7 points) indicate a higher risk of malnutrition.

The scales assessing the risk of falls by Runge and Rehfeld [17] and Oliver et al. (STRATIFY) [18] were used as screening instruments for difficulties in mobility associated with a risk of falls. The German Runge and Rehfeld scale consists of 10 items that are given weights of one or two points (e.g. disorders of gait and balance, reductions in the strength of the lower extremities, multimedication and taking medications associated with a risk of falling, a positive fall history, cognitive impairment with psychomotor restlessness); see additional material (Additional file 2: Scale) for the translated version. The STRATIFY-Fall-Risk-Assessment-Tool uses five items (e.g. recent history of falls, agitation, frequent toileting) with dichotomous answers yes/no (1 or 0 points). Higher scores (0–15 points for Runge and Rehfeld; 0–5 points for STRATIFY) indicate a higher risk of imminent falls.

The SF-36 [19] and EQ-5D-3 L [20] are widely used, validated instruments for the assessment of health-related quality of life (HRQoL) [21]. The raw scores of the 36 items from the SF-36 were transformed into standardised physical and mental component summary scores (short form: physical and mental scores), each of which ranges from 0 to 100. Also, the five items from the EQ-5D (we used the version with three levels of severity: 3L) were transformed into a standardised score ranging from 0 to 100. We used the “European Index”, which is based on large population-based samples from different European countries [22]. Higher scores on the SF-36 or EQ-5D indicate a higher HRQoL. Dorman et al. calculated test-retest reliability Kappa values ranging between 0.63 (anxiety/depression) and 0.80 (mobility) for a 3-week observation of stroke patients [23]. The differences between scores at discharge and the corresponding scores at admission represent the change in the parameter during inpatient rehabilitation. These changes were calculated for the SF-36 and EQ-5D.

### Statistical procedures

In a first step, bivariate analyses were computed to identify possible independent variables that were significantly correlated with HRQoL after one and 2.5 years, respectively (see Additional file 3: Table S1). Pearson correlations were computed to determine the associations between interval-scaled and dichotomous variables and the EQ-5D Index. Eta coefficients were calculated for nominal variables with more than two levels.

In order to identify the variables that predicted HRQoL in the multivariate context, we employed multiple linear regression analyses with the EQ-5D Indexes after one and 2.5 years as dependent variables. The non-standardised regression coefficient B and the respective 95 % confidence intervals are presented for each potential predictor.

The regression analysis was carried out in two blocks. Block I included the adjustment variables age, sex, and the EQ-5D Index at the time of discharge (enter method). This procedure makes it possible to interpret predictors of long-term HRQoL that are subsequently calculated independently of the patient’s age and sex, and above all, independently of the patient’s HRQoL at the time of discharge.

In block II, all other variables that were significantly associated (*p* < 0.05) with the outcome in the bivariate analyses were included in the regression model one at a time using stepwise forward selection until no significant variables were left. The threshold value for variable entry was *p* = 0.05, whereas *p* = 0.10 was used to remove a variable from the regression model.

However, to avoid multicollinearity in block II, we included only those independent variables that were not moderately or highly correlated with each other. When two independent variables were correlated at *r*(Pearson) ≥ 0.50, the variable with the higher bivariate correlation with the EQ-5D Index after 2.5 years was included in the regression analysis. The other variable had to be excluded due to multicollinearity. The two scales assessing the risk of falls were correlated *r*(Pearson) = 0.74; hence, the scale by Runge and Rehfeld was included in the analysis. The SF-36 mental component summary score (mental score) at discharge was associated with the change in the mental score between admission and discharge (*r* = 0.67); hence, the change in the mental score was included in the regression analysis. The SF-36 physical component summary score (physical score) at discharge was significantly correlated with the EQ-5D Index at discharge (*r* = 0.68). Thus, the EQ-5D Index at discharge was left in the analysis (block I).

In a sensitivity analysis, we investigated which predictors were associated with the individual dimensions of the EQ-5D. The main analysis model was modified in such a way that the individual EQ-5D dimension was employed instead of the EQ-5D Index. Binary logistic regression analyses were carried out by dichotomising the EQ-5D dimensions. A code of 0 was applied when there were no problems in the respective dimension; a code of 1 was given when there were problems.

Because we computed two multiple regression analyses (for the EQ-5D after one and 2.5 years), we applied a Bonferroni correction to adjust the significance level to *p* = 0.025. P-values between 0.025 and 0.05 were considered to be statistical trends. All statistical analyses were performed using the IBM SPSS® Statistics 21 package.

## Results

### Patients

The cohort consisted of 152 stroke patients who were discharged after a mean of 57 days (SD = 28 days) of inpatient neurological rehabilitation. Their mean age was 67.4 years (SD = 11.1), and 40 % were women (Additional file 3: Table S1). A total of 124 patients (82 %) had had an ischaemic stroke, 24 (16 %) an intracerebral haemorrhage, two (1 %) a subarachnoid haemorrhage, and two (1 %) a cerebral or sinus venous thrombosis.

The mean EQ-5D Index improved significantly between the time of admission and the time of discharge (*p* < 0.001) (see Additional file 4: Figure S2). This was evident at the level of the EQ-5D dimensions. In all five dimensions, the number of patients who improved was clearly greater than the number of patients whose condition deteriorated. The greatest difference between the number of improvements and the number of deteriorations was observed in the dimension “self-care” and the smallest in “pain/discomfort” and “anxiety/depression”. An additional file shows this in more detail (see Additional file 5: Table S2).

The mean indexes for the patients who were still at home 2.5 years after discharge remained at the same level between the time of discharge and follow-up (see Additional file 4: Figure S2). On the dimensional level, we found a more differentiated picture. In the period between discharge and 1 year later for half of the patients, changes were in fact evident on each of the EQ-5D dimensions–61 % in “usual activities” and 37 % in “self-care”. In the 18 months that followed, the number of changes fell to between 21 and 42 % (see Additional file 5: Table S2). In the follow-up period, there were more improvements than deteriorations on the “mobility” and “usual activities” dimensions, whereas on the “anxiety/depression” and “self-care” dimensions, the ratio of improvements to deteriorations remained roughly the same. On the “pain/discomfort” dimension, the number of deteriorations was twice as high as the number of improvements.

In both of the two multivariate regression analyses with the EQ-5D Indexes after one and 2.5 years as the dependent variable, respectively, it was found that, out of the three adjustment variables, only the EQ-5D value at discharge was significantly correlated with the dependent variable after 1 year (*p* = 0.017) and showed a statistical trend after 2.5 years (*p* = 0.049), (see Additional file 6: Table S3). Thus, the higher the HRQoL at discharge, the higher it was after one and 2.5 years.

In block II, a significant predictor of the EQ-5D Index after 1 year was identified, i.e. the value on Runge and Rehfeld’s risk of falls scale (*p* = 0.011). After 2.5 years, there was even an increase in the predictive strength of the risk of falls scale (*p* = 0.001). Thus, the higher the risk of falls upon admission to inpatient rehabilitation, the lower the HRQoL one and 2.5 years after discharge. The improvement in the SF-36 mental score during inpatient rehabilitation proved to be a predictor of HRQoL on the EQ-5D 2.5 years after discharge at the level of a statistical trend (*p* = 0.048).

In block II, functional independence (BI) at discharge (*p* = 0.31 (1 year) and *p* = 0.21 (2.5 years)), the risk of malnutrition (Nutritional Risk Score) (*p* = 0.19 (1 year) and *p* = 0.23 (2.5 years)), the comorbidity-based risk of mortality (Charlson Index) (*p* = 0.39 (1 year) and *p* = 0.08 (2.5 years)), and dysphagia upon admission to inpatient rehabilitation (*p* = 0.56 (1 year) and *p* = 0.06 (2.5 years)) were found to be non-significant.

The sensitivity analysis revealed that the change in the SF-36 mental score between admission and discharge from neurological inpatient rehabilitation was a significant (*p* = 0.004) predictor of the EQ-5D dimension “mobility” 2.5 years after discharge and showed a trend towards predicting “usual activities” (*p* = 0.05). Changes in the SF-36 mental score did not predict “self-care” (*p* = 0.08), “pain/discomfort” (*p* = 0.26), or “anxiety/depression” (*p* = 0.48). The risk of falls was significantly correlated with “mobility”, “self-care”, “usual activities” (all ps < 0.001), and “pain” (*p* = 0.010), but was not significantly correlated with “anxiety/depression” (*p* = 0.38).

In a further sensitivity analysis, we found that patients with obesity did not have significantly (*p* = 0.58) lower values on the EQ-5D than the other patients (63.3 (SD = 24.5) vs. 65.9 (SD = 24.9)).

We used two instruments to assess HRQoL in stroke survivors: the EQ-5D and the SF-36. The convergent validities between the EQ-5D Index measured at admission and discharge and the respective physical score on the SF-36 were significant and moderately strong: admission: *r*(Pearson) = 0.60, *p* < 0.001; discharge: *r*(Pearson) = 0.68, *p* < 0.001. The EQ-5D Index scores were also significantly correlated with the SF-36 mental scores, although the magnitudes were lower (*r*(Pearson) = 0.35 upon admission; *r*(Pearson) = 0.43 at discharge).

We were able to demonstrate the discriminant validity of the EQ-5D for use with stroke patients by computing the EQ-5D scores across the different levels of functional deficits as determined by the Barthel Index (BI). The mean EQ-5D Index for patients with mild impairment at discharge (BI > 90) was 74.4 (SD = 21.6). For patients with moderate impairment (BI 65–90), it was 71.4 (SD = 14.6), and for those with substantial impairments (BI < 65), it was 50.8 (SD = 21.2).

## Discussion

We measured HRQoL in stroke survivors in a single-centre cohort study, administering the EQ-5D and SF-36 upon admission to and at discharge from inpatient neurological rehabilitation. The EQ-5D was also administered by telephone one and 2.5 years after discharge.

During the inpatient neurological rehabilitation, HRQoL as assessed with the EQ-5D improved by a mean of one fifth of the range, from 45 to 67. This can be interpreted as resulting from the therapeutic effects of the rehabilitation and the spontaneous course. In order to be able to differentiate between these effects, we would have had to conduct a study with a control group that did not receive rehabilitation. However, this would have been unethical.

On average, for the stroke patients who were still living at home 2.5 years after discharge, HRQoL measured with the EQ-5D remained the same. Algurén et al. even observed a slight increase in HRQoL in 99 stroke patients following discharge from inpatient rehabilitation, measured with the Visual Analogue Scale of the EQ-5D. The mean value increased from 66 (6 weeks post-stroke) to 68 (3 months) and finally to 70 (after 1 year) [6]. In a population-based study of over 700 stroke survivors in Great Britain, Luengo-Fernandez et al. demonstrated that on average, the EQ-5D values remained stable in the period between 1 month and 5 years post-stroke [7].

In order to optimise the provision of health services and therapeutic interventions for stroke survivors after the acute treatment phase, we needed suitable parameters for measuring the quality of the outcome of the interventions. This function was fulfilled by the EQ-5D variables that predicted patients’ long-term progress as the EQ-5D unites essential aspects of subjective health.

Baseline values affect final values, and this of course applies to the EQ-5D. The results of the regression analyses showed that the EQ-5D values at discharge from inpatient rehabilitation (baseline) had a greater impact on the EQ-5D 1 year after discharge than on the EQ-5D 2.5 years after discharge. Thus, the predictive influence of the EQ-5D on HRQoL seems to decrease as time goes by. The results of the population-based study by Luengo-Fernandez et al. are consistent with this finding, as these authors also found high EQ-5D values after 5 years when the values at 1 month post-stroke had been high [7].

Irrespective of the EQ-5D, we found two significant predictors of HRQoL in the multivariate analysis. These were the risk of falls determined during inpatient rehabilitation and the change in emotional quality of life between admission and discharge.

The greater the risk of falls, the worse the HRQoL 2.5 years following discharge, especially on the EQ-5D dimensions “mobility”, “self-care”, and “usual activities”. The risk of falls scale constructed by Runge and Rehfeld includes different risks for falls, e.g. disorders of gait and balance, reductions in the strength of the lower extremities, multimedication and taking medications associated with a risk of falling, a positive fall history, and psychomotor restlessness with cognitive impairment play a major role [17]. Some of these factors are susceptible to intervention, e.g. gait and balance training, coordination and strength training, and a critical appraisal of medication. A review conducted by Gillespie et al. in community-dwelling older people revealed that physiotherapeutic training can reduce not only the risk of falls but also the actual frequency of falls [24]. For stroke patients, stable balance and mobility, which are part of functional status, lead to a better health-related quality of life [25].

We also found that the change–discharge score minus admission score–in emotional quality of life as depicted by the SF-36 during inpatient rehabilitation, but not the mental score at discharge, showed a trend towards predicting the EQ-5D Index after 2.5 years. The sensitivity analysis showed that this change in emotional quality of life during inpatient rehabilitation affected only the “body-related” dimensions of the EQ-5D after 2.5 years, especially mobility. Thus, it appears to be the case that stroke survivors with greater improvements in their emotional quality of life during rehabilitation rated their physical mobility more positively 2.5 years after discharge. Emotional well-being as part of the emotional quality of life measured by the EQ-5D is influenced in particular by the emotions “anxiety” and “depression” (The EQ-5D “anxiety/depression” item). Following a stroke, the occurrence of anxiety and depression is highly clinically significant (e.g. [26–28]). This suggests that the importance of anxiety and depression for long-term physical mobility should therefore not be underestimated, also with regard to setting goals for interventions. The reduction in the risk of falls and the improvements in emotional quality of life would appear to be suitable outcome parameters for making decisions about interventions for stroke survivors following acute treatment. The next step is to test the results of our correlational analyses as hypotheses for intervention studies.

Functional status also plays a central role in the assessment of stroke patients’ health. In their multivariate regression analysis of 77 cases, Haacke et al. found that the Barthel Index significantly predicted HRQoL as measured by the EQ-5D 4 years post-stroke [9]. Our study failed to verify this result for the 2.5-year period. This applies both to the level of the Barthel Index at discharge and to the change that occurred during the hospital stay. Our results indicate that the Barthel Index is not powerful enough to predict the long-term course of HRQoL after inpatient stroke rehabilitation.

The comorbidity-based mortality risk did not predict HRQoL in stroke patients still living at home after 2.5 years. This can be explained by the fact that the stroke survivors living at home are a positively selected group who had not died and had not been institutionalised. As expected, the comorbidity index proved to be a significant predictor of morbidity or institutionalisation [29].

If clinical conclusions are to be drawn on the basis of the EQ-5D, the measure must demonstrate validity. The EQ-5D’s validity was documented in Haywood et al.’s review article. The authors recommend that the EQ-5D should be used mainly for “patients in whom a substantial change of health is expected” [21]. Especially when using the EQ-5D with stroke patients, our study revealed a high convergent validity between the EQ-5D Index upon admission and at discharge and the respective physical score on the SF-36. This confirms Dorman et al.’s results [30]. The convergent validity between the EQ-5D Index and the SF-36 mental score was also significant but not as strong. Dorman et al. suggest that this difference may be due to differences in scale construction. Whereas the only mental aspects included in the EQ-5D are “anxiety” and “depression”, the SF-36 mental score also includes positive emotions [30]. Dorman et al. also investigated the validity of the EQ-5D for stroke patients on a dimensional level by using a separate assessment instrument for each EQ-5D dimension. The associations were 0.35 for “anxiety”, 0.56 for “depression”, and 0.71 for “pain” [31].

Does the EQ-5D also have discriminant validity when used with stroke patients? Whynes et al. investigated this question and demonstrated that the EQ-5D is good at differentiating between the levels of the Modified Rankin Scale (mRS). Patients who had no symptoms or significant disability after stroke (mRS 0 or 1) had EQ-5D Indexes of over 0.85, and patients with moderately severe difficulty in walking and attending to bodily needs (mRS 4) obtained indexes of 0.3 [32]. In our study, the EQ-5D could also be used to distinguish between stroke patients with different functional deficits measured with the Barthel Index (BI). The mean EQ-5D Index for patients with mild functional impairment (at discharge) was higher than the mean score for patients with moderate functional impairment, and this second score in turn was higher than the score for patients with substantial impairments. Therefore, we suggest that HRQoL declines as patients’ functional status deteriorates. Further studies are needed to establish the EQ-5D as an outcome criterion for intervention studies. For example, it is currently unclear how much the EQ-5D Index must improve to induce a clinically relevant change in HRQoL that the patient also esteems as a qualitative improvement.

The generalisability of our results is limited by the fact that they are based on a single-centre cohort of 152 stroke survivors. The very low drop-out rate during the observation period of 2.5 years can be considered one of our study’s strengths. Previous studies that have determined quality of life in stroke patients using the EQ-5D have had observation periods of only 3 to 12 months post-stroke (e.g. [6, 33, 34]). Moreover, in the regression analyses that we conducted to determine the predictors of HRQoL, we included clinical risk assessments for malnutrition, comorbidities, the risk of falls, and longitudinal data on changes occurring during inpatient rehabilitation.

On the basis of our results, we can derive two hypotheses that have not yet been tested in prospective intervention studies. We predict that patients’ health-related quality of life can be improved in the long-term following stroke if first, the risk of falls is reduced at an early stage, and second, anxiety and depressiveness are diagnosed and sufficiently treated.

## Additional files

Additional file 1: Figure S1. Consort flowchart. Additional file 2:
**Runge and Rehfeld’s risk of falls scale.**
Additional file 3: Table S1. Patient characteristics and bivariate analyses. Additional file 4: Figure S2. Longitudinal progression of HRQoL. Additional file 5: Table S2. Frequencies of the dimensions of the EQ-5D. Additional file 6: Table S3. Multivariate logistic regressions with the EQ-5D (European Index) 1 year and 2.5 years after discharge as the dependent variables.

## Abbreviations

ADL: Activities of daily living
BI: Barthel Index
BMI: Body mass index
CI: Confidence interval
EBI: Extended Barthel Index
EQ-5D-3L: EuroQol 5 dimensions 3 levels
ERBI: Early Rehabilitation Barthel Index
HRQoL: Health-related quality of life
mRS: Modified Rankin Scale
p: p-value
*r*(Pearson): Pearson correlation coefficient
SD: Standard deviation
SF-36: Short form 36
t: Test statistic
TOAST: Trial of Org 10172 in Acute Stroke Treatment
WHO: World Health Organisation
