# Supplementary material for: Predictors of health-related quality of life in stroke patients after neurological inpatient rehabilitation: a prospective study
Source: Health Qual Life Outcomes. 2015 May 14;13:58. doi: 10.1186/s12955-015-0258-9 (PMC4448207; doi:10.1186/s12955-015-0258-9)
Supplement: Additional file 1: Figure S1. — Consort flowchart. [file 12955_2015_258_MOESM1_ESM.pdf]

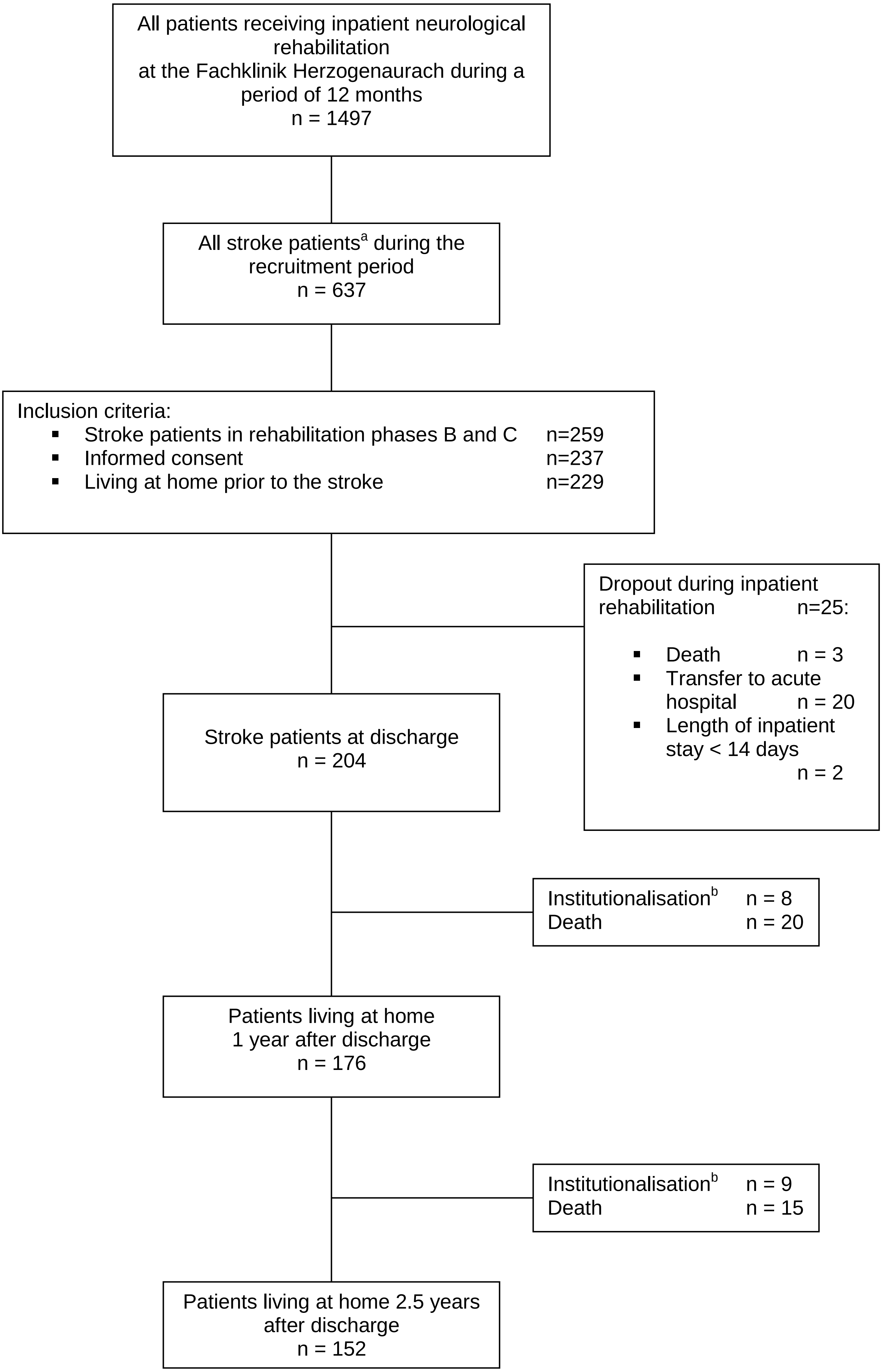
All patients receiving inpatient neurological rehabilitation
at the Fachklinik Herzogenaurach during a period of 12 months
n = 1497
All stroke patients[a] during the recruitment period
n = 637
Inclusion criteria:
Stroke patients in rehabilitation phases B and C n=259
Informed consent n=237
Living at home prior to the stroke n=229
Dropout during inpatient rehabilitation n=25:
Death n = 3
Transfer to acute hospital n = 20
Length of inpatient stay < 14 days n = 2
Stroke patients at discharge
n = 204
Institutionalisation[b] n = 8
Death n = 20
Patients living at home
1 year after discharge
n = 176
Institutionalisation[b] n = 9
Death n = 15
Patients living at home 2.5 years after discharge
n = 152

[a] Stroke according to WHO definition: ischemic stroke, intracerebral haemorrhage, subarachnoid haemorrhage, cerebral or sinus venous thrombosis

[b] Nursing home admission
