# Supplementary material for: Predictors of health-related quality of life in stroke patients after neurological inpatient rehabilitation: a prospective study
Source: Health Qual Life Outcomes. 2015 May 14;13:58. doi: 10.1186/s12955-015-0258-9 (PMC4448207; doi:10.1186/s12955-015-0258-9)
Supplement: Additional file 2: — Runge and Rehfeld’s risk of falls scale. [file 12955_2015_258_MOESM2_ESM.pdf]

Patient characteristic	Explanation	Points
**Disorders of gait, e.g.**<br>▪ unsteady gait<br>▪ spontaneous walking speed very slow (< 0.6 m/sec)<br>▪ abnormal score on the Tinetti test (gait score)<br>or<br>**Disorders of balance, e.g.**<br>▪ 10 sec tandem position not possible<br>▪ tandem gait not possible or unsteady<br>▪ marked unsteadiness or more than 12 steps in a 360° turn on the spot<br>▪ abnormal score on the Tinetti test (balance score)	**Clinical assessment**: steps small and irregular. Increased swaying of the body, deviations from the gait line, step height reduced. Frequent stumbling/ staggering, moves hand over hand/ grasps for support, stops when speaking. Core characteristic: increased variability in step sequence, the individual consecutive steps differ from each other in an irregular manner.<br><br>**Tandem position**: 3 attempts permitted. Feet aligned on a line one behind the other, the heel of the front foot touching the tips of the toes of the one behind. Arm position optional. Help in assuming the position permitted. "Tandem gait unsteady" means: 8 errors or more in 2 metres.	**2**
**Reduced strength – hip, knee or upper ankle joint**<br>▪ unable to rise from a chair without using arms to help or<br>▪ > 2 sec required per rise with 5 repeats (chair rising) or<br>▪ clinical strength test abnormal	**Chair rising**: Patient sits on a chair of conventional height (46 cm) and is asked to cross his/her arms across the chest and then stand up and sit down again immediately as fast as possible 5 times in a row.	**2**
**> 4 different medications**<br>**or**<br>**specific medications with evidence based association with a risk of falls**	Multimedication is likely to be an indicator of a general health impairment.<br>The following groups of medications increase the risk of falls: neuroleptics, tricyclic antidepressants, benzodiazepines with half-lives > 24h, anticonvulsants.	**2**
**Positive fall history**	3 or more non-syncopal falls a year or 1 fall with severe injury.	**2**
**Cognitive impairment with psychomotor restlessness that affects daily living**	Clinically evident as attention disturbance or psychomotor abnormality, restless pacing back and forth or gross errors in self-rating.	**2**
**Impaired vision**	Variously operationalised, e.g. generally as "affecting daily living" or at least 20 % vision loss. Pronounced differences in visual acuity between the eyes are particularly associated with falls.	**1**
**ADL deficits**<br>**or**<br>**the use of walking aids**<br>**or**<br>**progressive locomotor deterioration**	Operationalised as a personal need for support for one of the basic ADLs (ADLs = activities of daily living)<br><br>A history of, e.g. an increasing reduction in the daily locomotor radius	**1**
**Problems/ findings in the lower extremities with relevance for functionality**	E.g. chronic or sudden shooting pain, painful osteoarthritis, contractures, muscular atrophy, painful findings in the foot	**1**
**Parkinson's disease**<br>**or, in women,**<br>**hyperthyroidism in the history**	Those nosological diagnoses that are most frequently and most strongly associated with the risk of falls, irrespective of specific identifiable sequelae of illness	**1**
**85 years or older, or a low body mass index. Or, in women: weight less than 45 kg or failure to gain weight since the age of 25 or mother with hip fracture**	Some predictors of falls and fall-induced hip fracture have only been investigated in populations of women	**1**

≥ 4 points indicate a high likelihood of falling and an imminent risk of falls.
