# Supplementary material for: Predictors of health-related quality of life in stroke patients after neurological inpatient rehabilitation: a prospective study
Source: Health Qual Life Outcomes. 2015 May 14;13:58. doi: 10.1186/s12955-015-0258-9 (PMC4448207; doi:10.1186/s12955-015-0258-9)
Supplement: Additional file 3: Table S1. — Patient characteristics and bivariate analyses. [file 12955_2015_258_MOESM3_ESM.pdf]

Table 1 Patients' characteristics and bivariate analysis

Independent variable	Cohort n = 152 Mean (SD) or n (n%)	Correlation with dependent variable EQ-5D (European Index) 1 year after discharge r_p $(p)^a$	EQ-5D (European Index) 2.5 years after discharge r_p $(p)^a$
Sociodemographic variables			
Age (in years)	67.4 (11.1)	-0.01 (0.93)	-0.05 (0.51)
Sex (female)	60 (39.5%)	-0.11 (0.16)	-0.12 (0.14)
Clinical variables			
Rehabilitation phase (B)[b]	18 (11.8%)	0.07 (0.36)	0.04 (0.59)
BI at discharge	73.0 (16.6)	0.27 (0.001)*	0.31 (<0.001)*
BI change[c]	30.6 (15.2)	0.04 (0.66)	0.09 (0.27)
EBI at discharge	17.5 (5.3)	0.09 (0.29)	0.19 (0.019)*
EBI change[c]	3.4 (4.2)	-0.08 (0.36)	0.09 (0.28)
ERBI at admission[d]			
- Communication disorder	18 (11.8%)	-0.02 (0.84)	0.03 (p=0.70)
- Dysphagia	21 (13.8%)	-0.18 (0.028)[#]	-0.25 (0.002)*
ERBI at discharge[e]			
- Communication disorder	9 (5.9%)	-0.07 (0.40)	-0.04 (0.60)
- Dysphagia	9 (5.9%)	-0.09 (0.25)	-0.09 (0.27)
Body mass index	27 (4.3)	0.03 (0.75)	0.004 (0.96)
Nutritional Risc Screening	2.1 (1.8)	-0.16 (0.048)[#]	-0.16 (0.06)
Scale assessing risk of falls:			
- Runge and Rehfeld (1998)	5.2 (3.1)	-0.38 (<0.001)*	-0.45 (<0.001)*
- Oliver et al. (STRATIFY, 1997)	1.0 (1.1)	-0.21 (0.010)*	-0.24 (0.003)*
Stroke yes/no		0.05 (0.54)	-0.03 (0.74)
- yes	124 (81.6%)		
TOAST classification[f]		-0.02 (0.87)	-0.12 (0.17)
- Atherosclerosis	14 (11.3%)		
- Cardiac embolism	22 (17.7%)		
- Microangiopathy	38 (30.6%)		
- Other aetiology/ unknown aetiology	50 (40.3%)		
Ischemic stroke location		0.25 (0.003)*	0.18 (0.030)[#]
- unilateral right	59 (40.7%)		
- Unilateral left	74 (51.0%)		
- bilateral	12 (8.3%)		
Charlson Index	2.8 (1.2)	-0.11 (0.20)	-0.18 (0.027)[#]
Health-related quality of life			
SF-36 $_p$ at admission	30.5 (8.0)	0.30 (<0.001)*	0.29 (<0.001)*
SF-36$_p$ at discharge	37.3 (9.0)	0.33 (<0.001)*	0.26 (0.001)*
SF-36$_m$ at admission	39.1 (9.9)	-0.04 (0.61)	-0.10 (0.24)
SF-36$_m$ at discharge	45.4 (10.7)	0.15 (0.06)	0.23 (0.005)*
SF-36$_p$ change[c]	6.8 (9.2%)	0.06 (0.45)	0.001 (0.99)
SF-36$_m$ change[c]	6.3 (13.0%)	0.16 (0.05)	0.26 (0.001)*
EQ-5D at discharge	66.7 (19.5)	0.38 (<0.001)*	0.42 (<0.001)*
EQ-5D change[c]	21.4 (25.2%)	0.10 (0.21)	0.15 (0.07)

Abbreviations: *SD* , Standard deviation; *n (n%)* , absolute frequency (relative frequency); r_p, Pearson correlation coefficient; *BI* , Barthel Index; *EBI* , Extended Barthel Index; *ERBI* , Early Rehabilitation Barthel Index; *SF-36* $_p$, SF-36 physical component summary score; *SF-36* $_m$, SF-36 mental component summary score; *EQ-5D* , EuroQol 5 dimensions.

[a] Significant at $p < 0.025$ indicated by *; p-values = 0.025 - 0.05 as a statistical trend indicated by [#]

[b] Patients in phase B in neurological rehabilitation (all other patients phase C)

[c] Discharge score minus admission score

[d] Others criteria of the Early Rehabilitation Barthel Index at admission:
intensive care treatment 2 (1.0%), tracheostomy 0 (0.0%), artificial respiration 1 (0.5%), disorientation 9 (4.4%), behavioural disorders 6 (2.9%)

[e] Other criteria of the Early Rehabilitation Barthel Index at discharge:
intensive care treatment 0 (0.0%), tracheostomy 0 (0.0%), artificial respiration 1 (0.5%), disorientation 4 (2.0%), behavioural disorders 3 (1.5%)

[f] Aetiological classification of ischemic stroke
