# Supplementary figures and images for: Predictors of health-related quality of life in stroke patients after neurological inpatient rehabilitation: a prospective study
Source: Health Qual Life Outcomes. 2015 May 14;13:58. doi: 10.1186/s12955-015-0258-9 (PMC4448207; doi:10.1186/s12955-015-0258-9)

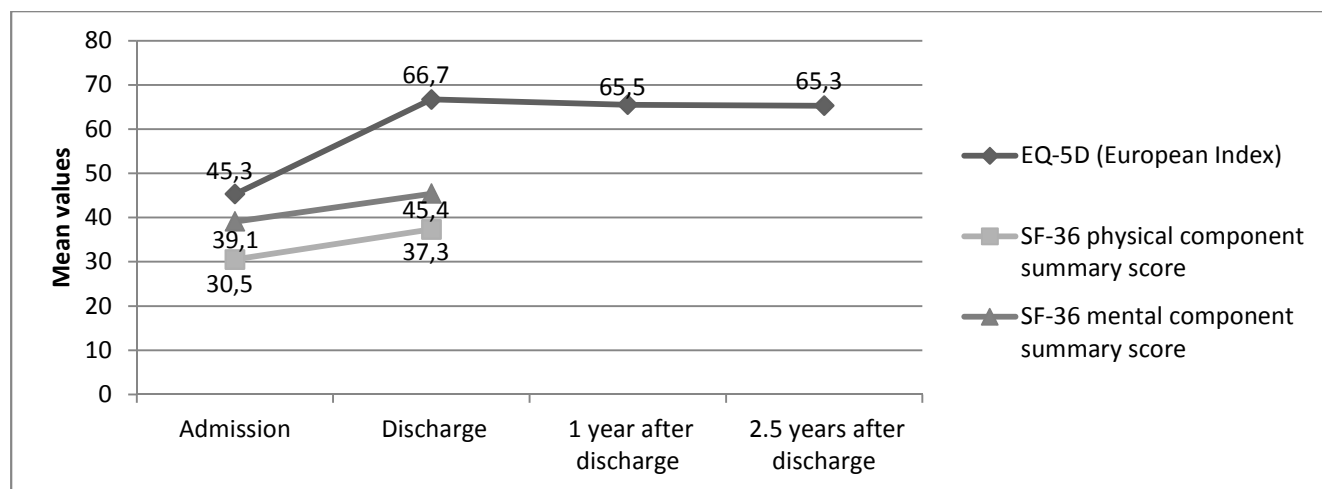

Supplement: Additional file 4: Figure S2. — Longitudinal progression of HRQoL. [file 12955_2015_258_MOESM4_ESM.pdf]
