# Supplementary material for: Predictors of health-related quality of life in stroke patients after neurological inpatient rehabilitation: a prospective study
Source: Health Qual Life Outcomes. 2015 May 14;13:58. doi: 10.1186/s12955-015-0258-9 (PMC4448207; doi:10.1186/s12955-015-0258-9)
Supplement: Additional file 5: Table S2. — Frequencies of the dimensions of the EQ-5D. [file 12955_2015_258_MOESM5_ESM.pdf]

Table 2 Frequencies of the dimensions of the EQ-5D. *n* =142

Dimension	Admission[a] (*t1*)	Discharge[a] (*t2*)	*t1* - *t2*	1 year after discharge (*t3*)	*t2* - *t3*	2.5 years after discharge (*t4*)	*t3* - *t4*
Mobility							
- no problems	24 (16.9%)	62 (43.7%)		90 (63.4%)		100 (70.4%)	
- some problems	101 (71.1%)	77 (54.2%)		50 (35.2%)		39 (27.5%)	
- confined to bed	17 (12.0%)	3 (2.1%)		2 (1.4%)		3 (2.1%)	
Improvement			57 (40.1%)		46 (32.4%)		22 (15.5%)
Deterioration			9 (6.3%)		17 (12.0%)		13 (9.2%)
Self-care							
- no problems	25 (17.6%)	79 (55.6%)		100 (70.4%)		98 (69.0%)	
- some problems	92 (64.8%)	56 (39.4%)		33 (23.2%)		36 (25.4%)	
- unable	25 (17.6%)	7 (4.9%)		9 (6.3%)		8 (5.6%)	
Improvement			77 (54.2%)		37 (26.1%)		14 (9.9%)
Deterioration			10 (7.0%)		16 (11.3%)		16 (11.3%)
Usual activities							
- no problems	6 (4.2%)	25 (17.6%)		54 (38.0%)		55 (38.7%)	
- some problems	74 (52.1%)	105 (73.9%)		47 (33.1%)		46 (32.4%)	
- unable	62 (43.7%)	12 (8.5%)		41 (28.9%)		41 (28.9%)	
Improvement			66 (46.4%)		44 (31.0%)		32 (22.5%)
Deterioration			6 (4.2%)		42 (29.6%)		24 (16.9%)
Pain/discomfort							
- none	53 (37.3%)	87 (61.3%)		87 (61.3%)		72 (50.7%)	
- moderate	84 (59.2%)	52 (36.6%)		49 (34.5%)		62 (43.7%)	
- extreme	5 (3.5%)	3 (2.1%)		6 (4.2%)		8 (5.6%)	
Improvement			51 (35.9%)		31 (21.8%)		22 (15.5%)
Deterioration			16 (11.3%)		33 (23.2%)		37 (26.0%)
Anxiety/depression							
- none	41 (28.9%)	74 (52.1%)		75 (52.8%)		70 (49.3%)	
- moderate	89 (62.7%)	63 (44.4%)		61 (43.0%)		63 (44.4%)	
- extreme	12 (8.5%)	5(3.5%)		6 (4.2%)		9 (6.3%)	
Improvement			54 (38.0%)		37 (26.1%)		25 (17.6%)
Deterioration			19 (13.4%)		37 (26.1%)		30 (21.1%)

Collection of the complete HRQoL data until 2.5 years after discharge from neurological inpatient rehabilitation by telephone interview with 142 patients.

[a] Admission to and discharge from neurological inpatient rehabilitation
