# Supplementary material for: Predictors of health-related quality of life in stroke patients after neurological inpatient rehabilitation: a prospective study
Source: Health Qual Life Outcomes. 2015 May 14;13:58. doi: 10.1186/s12955-015-0258-9 (PMC4448207; doi:10.1186/s12955-015-0258-9)
Supplement: Additional file 6: Table S3. — Multivariate logistic regressions with the EQ-5D (European Index) 1 year and 2.5 years after discharge as the dependent variables. [file 12955_2015_258_MOESM6_ESM.pdf]

Table 3 Multivariate logistic regressions with the EQ-5D (European Index) 1 year and 2.5 years after discharge as the dependent variables.

Independent variable	EQ-5D 1 year after discharge<br>Regression coefficient *B* (95% *CI*)	*t*	p^a	EQ-5D 2.5 years after discharge<br>Regression coefficient *B* (95% *CI*)	*t*	p^a
Block I [b]						
Age	0.04 (-0.27 - 0.34)	0.22	0.82	-0.05 (-0.37 - 0.27)	-0.31	0.76
Sex[c]	-4.32 (-11.23 - 2.63)	-1.23	0.22	-6.18 (-13.38 - 1.03)	-1.70	0.09
EQ-5D at discharge	0.27 (0.05 - 0.49)	2.42	0.017*	0.24 (0.001 - 0.47)	1.98	0.049#
Block II [d]						
BI at discharge	0.09	1.02	0.31	0.11	1.27	0.21
ERBI at admission, Dysphagia	-0.05	-0.58	0.56	-0.15	-1.91	0.058
Nutritional Risk Screening	-0.10	-1.31	0.19	-0.09	-1.20	0.23
Scale assessing risk of falls - Runge and Rehfeld	-1.77 (-3.14 - -0.41)	-2.57	0.011*	-2.31 (-3.70 - -0.92)	-3.28	0.001*
Charlson Index	-0.07	-0.86	0.39	-0.13	-1.79	0.08
$SF\text{-}36_m$ change[e]	0.05	0.61	0.54	0.29 (0.003 - 0.58)	1.99	0.048#

Abbreviations: *EQ-5D*, EuroQol 5 Dimensions; *CI*, Confidence interval, *t*, Test statistic, p, *BI*, Barthel Index; *ERBI*, Early Rehabilitation Barthel Index; $SF\text{-}36_m$, SF-36 mental component summary score.

[a] Significant at $p < 0.025$ indicated by *****; p-values = 0.025 - 0.05 as a statistical trend indicated by #

[b] Adjustment variables using enter method

[c] Code: female = 0, male = 1

[d] Final regression model (variables in the equation) using stepwise forward selection

[e] Discharge score minus admission score
